# The efficacy of photodynamic therapy in rat tongue dysplasia

**DOI:** 10.4317/jced.54425

**Published:** 2019-07-01

**Authors:** Faezeh Khozeimeh, Samaneh Ziaei, Saeedeh Khalesi, Maryam Allameh, Gholamreza Jahanshahi

**Affiliations:** 1 DDS, MS; Associate Professor, Dental Research Center, Dept. of Oral Medicine, Dental Research Institute, Isfahan University of Medical Sciences, Isfahan, Iran; 2 DDS, MS; Assistant Professor, Dept. of Oral and Maxillofacial Medicine, Faculty of Dentistry, Shahrekord branch, Shahrekord, Iran; 3 DDS, MS; Assistant Professor, Dental Material Research Center, Dept. of Oral and Maxillofacial Pathology,Dental Research Institute, Isfahan University of Medical Sciences, Isfahan, Iran; 4 DDS, MS; Assistant Professor, Dept. of Oral Medicine, Faculty of Dentistry, Shahed University, Tehran, Iran; 5 DDS, MS; Full professor of Oral Pathology, Faculty of Dentistry, Isfahan University of Medical Sciences, Isfahan, Iran

## Abstract

**Background:**

Photodynamic therapy (PDT) using 5-aminolevulinic acid (ALA) has previously shown promising results in cancerous cell destruction. The present study was conducted to evaluate the efficacy of this treatment option on oral epithelial dysplasia in Wistar rats. Furthermore, microscopic effects of systemic versus topical administration of ALA before laser illumination was assessed.

**Material and Methods:**

Thirty male Wistar rats (200- 250 grams) were used in the present study. Tongue dysplasia was induced by a daily delivery of a 20 ppm solution of 4-nitroquinoline -1- oxide (4NQO) for 3 months. Then, rats were divided into 3 groups of 10 including, group 1 that was received systemic ALA-based PDT (30 mg/kg ALA), group 2 that was received topical ALA-based PDT (20% ALA solution) and group 3 (control) which was left untreated. Tongue specimens were fixed for histopathological evaluation and dysplasia was graded at microscopic level. Data was compared between various treatment groups using Mann Whitney test (*p*<0.05).

**Results:**

The rate of atypical dysplastic cells was decreased significantly in both topical (*p*= 0.006) and systemic (*p*= 0.001) treatment groups compared to control group. Furthermore, systemic use of ALA resulted in a remarkable destruction of dysplastic cells compared to its topical application (*p*=0.045). Nevertheless, some evidence of muscle destruction was documented in systemic ALA group.

**Conclusions:**

It seems that ALA mediated PDT is an effective treatment option for the destruction of dysplastic cells. However, the extent of this effect depends on the mode of ALA administration before light illumination.

** Key words:**Photodynamic therapy, 5-aminolevulinic acid, Dysplasia, Potentially malignant disorders.

## Introduction

Oral epithelial dysplasia has been defined as the presence of architectural and cytological alterations with an increased likelihood of progression to cancer (1). This general term covers various stages of the disease from a mild alteration with basal/parabasal hyperplasia to carcinoma in situ (1). Currently, surgical removal is the accepted treatment modality for the management of these lesions, regardless of their grade (1,2). Excision of dysplastic area would significantly decrease the risk of cancer progression and recurrence rate (2,3). However, resection treatment is very painful and may require extensive reconstructive techniques regarding the vast areas of oral cavity that are affected (2). In spite of remarkable successes in the diagnosis and grading of epithelial dysplasia, there is no consensus on appropriate treatment choices for each stage (3). Therefore, exploring new treatment modalities against dysplastic cells with less invasive and more specific operations is of paramount importance. Photodynamic therapy (PDT) is a new FDA-approved anti-cancer modality (4) which employs three components of photosensitizer (PS), light and oxygen to produces free radicals in site of PS accumulation (5). The anti-tumor efficacy of PDT is illustrated by 3 mechanisms: 1) direct tumor cell destruction, 2) anti-vascular effects and 3) induction of inflammatory reactions (4). Many photosensitizers have been introduced since the advent of this drug-device modality; however, 5-aminolevulinic acid (ALA) has shown promising effects (6). The administration rout of ALA can be both topical and systemic (7). No evidence has so far been found to support any toxic or allergic effects for the systemic use of ALA. However, it has not determined that which kind of ALA administration is more efficient for the treatment of superficial lesions by PDT.

Recently, the treatment of non-melanoma skin cancer by ALA-PDT has been received much attention (7). It has been reported that potentially malignant lesions like actinic keratosis are the most sensitive lesions to this treatment modality (7); however, there is a lack of evidence concerning the efficacy of this treatment option on oral premalignant lesions. The present study was conducted to examine the efficacy of topical and systemic ALA-based PDT in a rat experimental model of tongue epithelial dysplasia.

## Material and Methods

-Animals

The study was conducted on male Wistar rats of 4 weeks old (200-250 grams). The protocol of the study was approved by ethics committee of Isfahan University of Medical Sciences for animal care and handling, Isfahan, Iran (Research project number:). The initial number of rats was 30; however, five extra rats were later added to replace animals which were excluded from the study. The animals were kept in separate cages under standard conditions of humidity and temperature. Easy access to food and water and a 12-hour light cycle were provided for all rats.

-Developing epithelial dysplasia in rats

The powder form of 4-nitroquinoline1-oxide (4NQO) (Sigma-Aldrich, USA) was added to rats’ water bottle for creating a 20 ppm solution. The water supply was filled with fresh water every three days. After 12 weeks, rats’ oral tissues, especially posterior lateral borders of the tongues, in the control group was inspected macroscopically to detect clinical changes such as leukoplakia or erythroplakia. The dissection of the tongues was performed following detection of tissue alterations based on clinical evidence. Then, histopathological assessment was carried out on both right and left sides of the tongues to confirm occurrence of dysplasia in epithelium. Upon confirmation, the consequent steps were performed on rats in the treatment groups. We did not limit ourselves to this confirmation in the control group and adhered to exclusion criteria in the treatment groups as well. According to the exclusion criteria, any rat with a dysplasia grade of less than moderate was excluded from the study and replaced by another case which was reserved for this purpose.

-PDT mediated systemic ALA

The rats in the group 1 (systemic ALA-based PDT group) received 30 mg/kg ALA (Sigma Aldrich, USA) through intramuscular injection. Treated animals were kept in a dark room for 4 hours and then exposed to light illumination. Rats were superficially anesthetized through inhalation of chloroform gas and a molt mouth gag was applied to open their mouth and take out their tongues for laser therapy. Then, visible light, with specifications which have summarized in  Table 1, was illuminated to the left posterior one third of lateral border of rats’ tonguesfor 5 minutes. Other parts of the tongues were covered by an aluminum foil to protect them from light emitting. One hour after the intervention, the rats were killed painlessly by excessive anesthetics and their tongues were removed and fixed in 10% formalin for further assays.

**Table 1:**
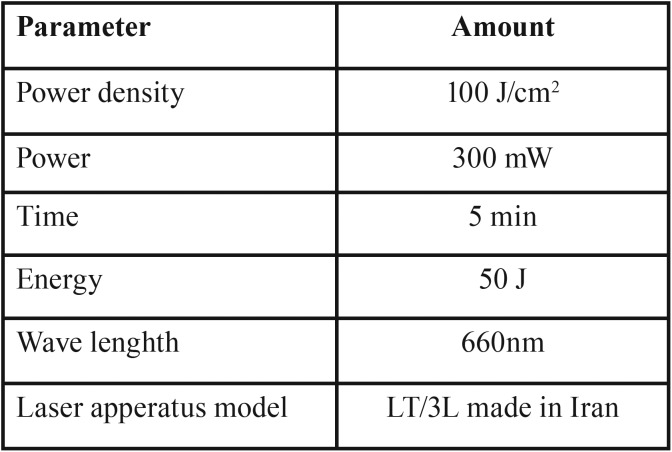
Laser parameters.

-PDT mediated topical ALA

An ALA solution with a concentration of 20% was prepared by dissolving ALA powder (Sigma Aldrich, USA) into ethylene glycol oil. Rats were superficially anesthetized and a swab which was impregnated with ALA solution was rubbed onto the posterior one third of lateral border of rats’ tongues. This procedure was repeated two hours later. Four hours after the first ALA application, the tongues were exposed to visible light (with the same characteristics as the light used for systemic ALA treatment group) for 5 minutes. Rats were killed by excessive use of anesthetics and their tongues were excised for fixation and histopathological assessment.

-Histopathology examination

Samples were fixed in formalin 10% and embedded in paraffin for histopathological assessments. Then sections with 5 µm thickness were stained by hematoxylin and eosin. Samples were assigned grades of 0-3 corresponding to dysplasia severity stage (normal, mild, moderate, sever) through a semi-quantitative grading approach for cytological changes (1). Additionally, any evidence of vessel modification, unusual immune cell infiltration or tissue alterations which was not associated with dysplasia progression was recorded by two blinded expert pathologists.

-Statistical analyzes

Cohen’s κ was run to determine if there was any disagreement between two pathologists. Mann Whitney test was used to compare stage scores between groups. Thevalue of *p*<0.05 was considered statistically significant.

## Results

There was a good agreement between two pathologists’ reports (κ = .893). Dysplasia was graded as mild in the right side of the tongue in 5 rats (3 in group 1 and 2 in group 2). As a result, they were excluded from the study and replaced by a reserved rat. Finally, a total of 35 rats were submitted to this investigation.

**Figure 1:**
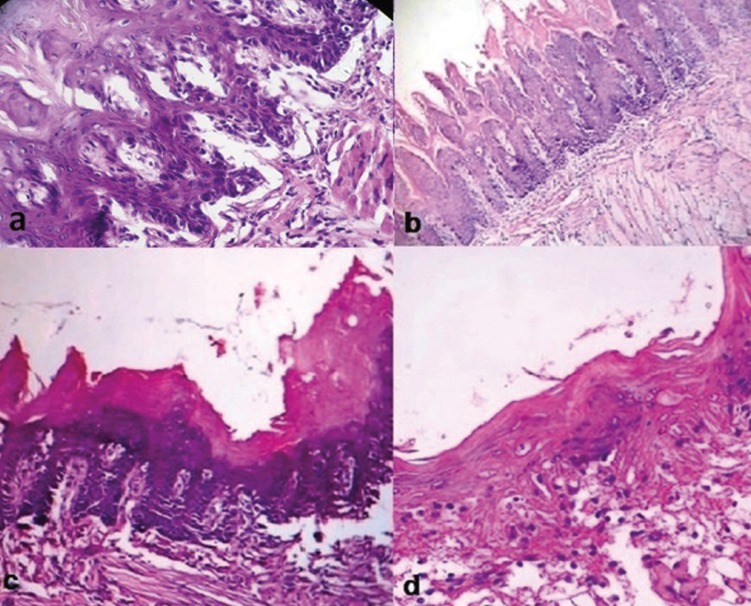
(a) Histologic appearance of tongue epithelium with severe dysplasia in a rat of control group (H&E staining, ×400). (b,c,d) Moderate dysplasia and hyperkeratosis in the rats of control group (H&E staining, ×40).

Qualitative assessment of samples in the control group showed a higher score of dysplasia in posterior areas of rats’ tongue. The grade of dysplasia in both sides of posterior lateral area of rats’ tongue was almost similar in the control group. Severe dysplasia was observed in lateral posterior one third of the tongue in seven rats of the control group (Fig. 1a) including one sample of carcinoma in situ. Three cases of moderate to severe dysplasia were also recorded (Fig. 1b-d). Clinical features in most cases were presented as erythroplakia; while, in some cases verrucous hyperplasia of epithelium was observed. After light illumination, the intervened tissue was warmer than other sites and its appearance in most cases was like a necrotic tissue with a white color. The results showed that topical and systemic ALA-based PDT treatment significantly reduced the rate of dysplasia (*p*<0.05) ( Table 2). Moreover, PDT-mediated systemic ALA administration showed more potency in the clearing of dysplastic cells than PDT induced by topical application of this photosensitizer (*p*=0.045).

**Table 2:**
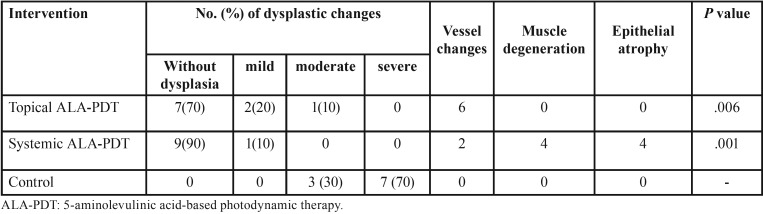
Microscopic results in two experimental groups compared to control.

Signs of cell degeneration in basal and hyper-basal levels were observed in the most sections of the treated areas of rats’ tongues in both experimental groups; however, atrophic epithelium was also observed in samples treated by systemic ALA.

**Figure 2:**
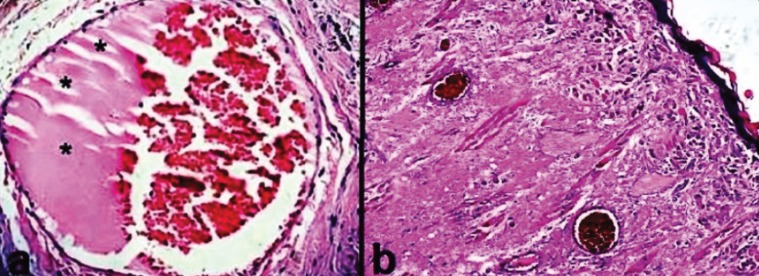
(a) Histologic view of amorphous deposits developed (asterisks) in cross section of blood vessels in groups treated with PDT (H&E staining, ×400). (b) Muscle degeneration in a sample employed to systemic ALA administration followed by laser (H&E staining, ×100).

An amorphous material was seen in microscopic assessment of vascular cross sections in two rats in the group 1 and six rats in the group 2 (Fig. 2a). Moreover, four cases in the group 1 showed evidence of muscle degeneration without any unusual infiltration of inflammatory cells (Fig. 2b). There was no sign of inflammatory reactions in two treatment groups.

## Discussion

In the current study, we assessed the treatment of carcinogen induced tongue dysplasia in rats with PDT modality in combination with systemic or oral ALA.

The method for dysplasia induction in animals was adapted from previous studies (10-13). Although, all of these studies have been used 4NQO exposure for SCC induction; in the current study, the use of this modality for about 3 months produced moderate to severe dysplasia in all rats in the control group. The approach was somewhat time consuming, but its efficacy was acceptable. To make sure of dysplasia development before the intervention, tongue tissues were assessed and those without any evidence of dysplasia were eventually eliminated from the study.

The results of the present study demonstrated that ALA-PDT was an effective treatment in tongue dysplasia. The systemic mode of photosensitizer administration was more efficient than its topical administration mode. ALA is a hydrophilic compound and the rational for its topical use relies on penetration of photosensitizer through defective cell membrane of tumor cells (8). In early stages of dysplasia, the altered cells are covered with deep layers of epithelium (1). This phenomenon can explain why topical application of the photosensitizer in the present study did not work as well as the systemic treatment approach. The photosensitizer was dissolved in an oil emulsion to create potentiated penetration in cells. However, it seems that the excess ALA could not find its way into deep tissue layers and deposited on the surface layer. As a result, the laser beams could not reach deeper layers. It is necessary to examine this hypothesis through fluorescence imaging in future studies. It has been suggested that repeated sessions of PDT mediated topical ALA would improve outcomes (7).

Up to date, no evidence exists against safety of systemic administration of ALA, except for occasional reports of skin reactions against this material, which has made to use anesthetic drugs routinely for patients receiving this treatment (9). In the present study, unexpected muscle degeneration was reported in four rats which were received systemic ALA. Further studies are needed to examine muscle degeneration in human subjects. Considering the nature of the present study, it was not possible to follow up the cases after treatment; however, according to Peng *et al.*, the assessment process should continue 1-2 months after termination of PDT sessions to evaluate the response in patients (7).

In ALA-mediated PDT, the duration of photosensitizer application before light exposure has of great importance. Because ALA can produce protopurphirin IX in tissues during a time of 4 hours or more (7). Accordingly, in the present study, topical application of ALA was carried out twice with a 2-hour interval, followed by light exposure 4 hours after the first ALA administration.

Present study showed 70% complete resolution of dysplastic tissues after topical administration of ALA and light emission; which is much less than the remission has been reported in previous published clinical studies (7). This may be due to different assessment procedures which have been applied in clinical studies compared to the present study. Complete resolution in clinical studieshas been determined upon clinical examinations in follow up sessions. However, microscopic assessment of resolution in the current study resulted in categorizing of atypical cells as dysplastic. Castano *et al.* reported that the outcome of PD is associated with the interaction between PS and cells of targeted tissue or tumor (8). In addition, it has been suggested that PS penetrates atypical cells more easily (8). As a result, it supposed that PS penetration improves in higher stages of dysplasia. This is also maybe illustrative for best results of PDT intervention in SCC compared to dysplasia subjects.

In the current study, the immediate features which were found in dysplastic tissues following PDT were apoptosis and cell destruction. The results of a study by Zaidi *et al.* were in direction with our findings (14).

In the present study, intact tissue panels or mild dysplasia were observed in treated tissues. It is suggested that they might be related to free radicals which are produced by PDT. Moreover, in systemic ALA-PDT group, epithelial atrophy was observed, which is due to the elimination of destroyed epithelial cells during the treatment process. Raised light dose in this treatment procedure improves tumor destruction; whereas, it is possible that it damages healthy tissues (15). Our findings showed that systemic ALA-PDT resulted in 90% (9 cases) complete resolution of dysplasia followed by some complications of muscle degeneration and epithelial atrophy. The results of a study by Fink-Puches *et al.*, indicated sever fibrosis in deep layers of BCC and SCC patients’ skin following the treatment with ALA-PDT (16). As a result, it is important to explore PDT side effects and preventive strategies for their management in future studies.

Agostinis *et al.* (4) proposed three mechanisms for PDT efficacy in cancer treatment, including cytotoxicity, anti-vascular proceed and immune cell induction. In our study, an amorphous material was found in cross sections of vessels. This was mainly observed in rats that received topical ALA-PDT treatment. More investigations are needed to identify the correlation between this observation and PDT effects. Our results demonstrated cell death and vessel changes after PDT which is in direction with the results of a study by Henderson et al (15). In the present study, PDT resulted in vessel occlusion; whereas, vessel destruction was observed in the study by Henderson *et al.* (15). The higher energy density of Laser in the study can explain the differences in vessel changes following topical ALA-PDT. The importance of these two studies mostly comes from the lack of augmented immune response after PDT. Previous investigations have suggested that one mechanisms by which PDT creates its antitumor activities is the induction of immune cells following tumor cell destruction (4,8). Undetectable evidence of augmented immune response is possibly logical in the present study because we could not follow our subjects like human subjects. Additionally, human responses to the treatment are more potentiated; as a result, immune cell recall after PDT implementation in human subjects is much easier compared to experimental animals.

The current protocol for dysplasia treatment did not cover all of expectations regarding high rate of relapse and discomfort in experimental animals following excision (1). The present study also assessed the rapid changes in dysplastic tissues undergoing PDT. As a result, considering promising results of the current study, it is recommended that future studies explore optimum PDT parameters for the treatment of dysplasia with minimum side effects thought evaluating the established effects of this intervention on tissues in later phases.

## Conclusions

The results of the study showed that, ALA-mediated PDT is a successful treatment modality against oral dysplasia. In addition, the systemic administration of ALA led to complete resolutions in more subjects compared to its topical administration. Further studies are needed to evaluate these results in human manifestation of dysplasia.
